# Case Report: Acute Pancreatitis in an Uncontrolled Diabetic Patient Presenting With a Skin Rash Later Found to Be Eruptive Xanthoma, a Dermatological Manifestation and Diagnostic Clue to Recognizing Hypertriglyceridemia as the Cause of Disease

**DOI:** 10.7759/cureus.64563

**Published:** 2024-07-15

**Authors:** Paul Q Vu, Mrudula Thiriveedi, Siddharth Patel, Rajesh Metuku

**Affiliations:** 1 Internal Medicine, Alabama College of Osteopathic Medicine, Dothan, USA; 2 Internal Medicine, Decatur Morgan Hospital, Decatur, USA

**Keywords:** generalized skin rash, eruptive xanthoma, diabetic ketoacidosis (dka), uncontrolled diabetes mellitus, nausea and vomiting, abdominal pain, hypertriglyceridemia-induced acute pancreatitis

## Abstract

Acute pancreatitis results from any insult that leads to inflammation of the organ. Hypertriglyceridemia is one of the risk factors associated with acute pancreatitis. The typical presentation is abdominal pain, nausea, and vomiting. We present a unique case in which the patient’s condition was further complicated by diabetic ketoacidosis. Consequently, he presented somnolent to the emergency room, providing a limited history only pertaining to generalized weakness and a skin rash. The patient was found to have hypertriglyceridemia-induced pancreatitis, which was appropriately managed in the intensive care unit. The skin lesions were diagnosed as xanthomas, which are associated with hypertriglyceridemia and acute pancreatitis secondary to hypertriglyceridemia. The patient was discharged on fibrate therapy, dietary counseling, and strict monitoring by his primary care physician. This unique case highlights the importance of recognizing dermatological conditions and their associated diseases to allow for prompt diagnosis and treatment in the face of limited history.

## Introduction

Pancreatitis is an inflammatory condition of the pancreas that can be classified as either acute or chronic. Acute pancreatitis is the leading gastrointestinal cause of hospitalization in the United States [1]. It can be further classified as mild acute (e.g., absence of organ failure), moderately severe (e.g., transient organ failure that resolves within 48 hours), or severe (e.g., persistent organ failure). While numerous causes can result in acute pancreatitis, cholelithiasis and alcohol-induced damage account for two-thirds of cases [2]. Another common etiology for acute pancreatitis is hypertriglyceridemia, and especially when serum triglyceride levels are over 1000 mg/dL, attacks of acute pancreatitis are more likely to occur [3]. The three common symptoms of hypertriglyceridemia-induced acute pancreatitis include abdominal pain, nausea, and vomiting [4]. We present an atypical case of hypertriglyceridemia-induced pancreatitis, where the initial symptoms did not involve abdominal pain, nausea, or vomiting but were a skin rash and generalized weakness.

## Case presentation

The patient is a 31-year-old male with a history of type 2 diabetes mellitus who has been completely non-compliant with his medications for one year and presents to the emergency room complaining of generalized weakness and a diffuse skin rash worsening over the past two weeks. Upon arrival, the patient was somnolent, and the initial history and physical examination were limited. Vitals showed a temperature of 97.4 °F, blood pressure of 136/84 mmHg, pulse of 115 per minute, respiratory rate of 22 per minute, and oxygen saturation of 100% on room air. Auscultation of the heart and lungs revealed tachycardia with a normal rhythm, no murmur, rub, or gallop, and mild tachypnea with normal breath sounds without wheezing, rhonchi, or crackles, respectively. Peripheral pulses were 2+, and no motor/sensory deficits were noted on neurological examination. His abdomen was soft, non-distended, and mildly tender to palpation in the epigastric region, with no guarding or rebound tenderness. Skin examination revealed diffuse papular lesions in the bilateral upper and lower extremities, face, and chest, which were irregular, firm, non-tender, and non-erythematous yellow-colored and nonpruritic. Initial laboratory studies, including complete blood count (CBC), complete metabolic panel (CMP), erythrocyte sedimentation rate (ESR), and C-reactive protein (CRP), were obtained. CBC was significant for leukocytosis (22 x 10^3^ cells/mm^3^), thrombocytosis (462 x 10^3^ cells/mm^3^), and elevated ESR and CRP of 100 mm/hour and 485 mg/L, respectively (Table 1).

**Table 1:** Complete blood count, coagulation studies, and inflammatory markers on admission

Test	Result	Reference Range
White blood cell count	21.25 x 10^3 ^cells/mm^3^	4.8-10.8 x 10^3^ cells/mm^3^
Red blood cell count	4.37 x 10^6^ cells/mm^3^	4.7-6.1 x 10^6^ cells/mm^3^
Hemoglobin	14.0 g/dL	14.0-18.0 g/dL
Hematocrit	39.7%	42.0-52.0%
Mean corpuscular volume	90.8 FL	81-99 FL
Mean corpuscular hemoglobin	32.0 PG	27-31 PG
Mean corpuscular hemoglobin concentration	35.3 g/dL	33-37 g/dL
Red cell distribution width-standard deviation	14.0%	11.5-14.5%
Platelet count	462 x 10^3^ cells/mm^3^	130-400 x 10^3^ cells/mm^3^
Prothrombin time	15.5 seconds	11.0-16.0 seconds
International normalized ratio	1.17	0.9-1.1
Partial thromboplastin time	33.9 seconds	22.3-41.8 seconds
Erythrocyte sedimentation rate	100 mm/hour	0-15 mm/hour
C-reactive protein	485 mg/L (Day 1); 97 mg/L (Day 5)	0.0-5.0 mg/L

With a limited history, a nonsignificant physical exam aside for skin lesions, and mild epigastric tenderness, the working diagnosis was to rule out an infectious process. The CMP hemolyzed a few times, prompting the nurse to personally obtain another sample, transport it to the lab, and have it immediately analyzed. The CMP showed a bicarbonate level of 4 mmol/L, elevated anion gap of 31 mmol/L, glucose level of 528 mg/dL, positive serum acetone, and urinalysis positive for glucose and ketones (Table 2).

**Table 2:** Comprehensive metabolic panel on Day 1 and Day 3 of hospitalization

Test	Results		Reference Range
	Day 1	Day 3	
Sodium	115 mmol/L	132 mmol/L	136-145 mmol/L
Potassium	5.6 mmol/L	3.1 mmol/L	3.5-5.1 mmol/L
Chloride	80 mmol/L	99 mmol/L	88-107 mmol/L
Bicarbonate	4 mmol/L	22 mmol/L	25-35 mmol/L
Anion gap	31 mmol/L	11 mmol/L	8-14 mmol/L
Blood urea nitrogen	37 mg/dL	4 mg/dL	8-22 mg/dL
Creatinine	1.2 mg/dL	0.5 mg/dL	0.7-1.2 mg/dL
Glucose	528 mg/dL	137 mg/dL	70-104 mg/dL
Calcium	8.8 mg/dL	8.4 mg/dL	8.8-10.2 mg/dL
Magnesium	2.3 mg/dL	1.7 mg/dL	1.5-2.7 mg/dL
Phosphorus	1.0 mg/dL	1.9 mg/dL	2.7-4.5 mg/dL
Total bilirubin	0.16 mg/dL	-	0.20-1.00 mg/dL
Aspartate aminotransferase	17	-	10-34 U/L
Alanine aminotransferase	<5 U/L	-	10-44 U/L
Alkaline phosphatase	158 U/L	-	32-122 U/L
Serum acetone	Large	-	Negative
Amylase	54 U/L	-	20-100 U/L
Lipase	66 U/L	-	13-60 U/L

**Figure 1:**
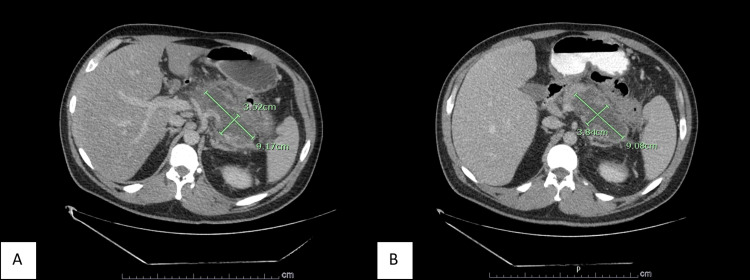
Transverse section CT scan of the abdomen/pelvis (A) Day 1: The pancreatic/peripancreatic inflammation is most prominent in the body and tail regions of the pancreas, with associated necrosis and pseudocyst formation at the pancreatic body and tail measuring 9.2 cm x 3.5 cm. (B) Day 5: Stable acute pancreatitis with a peripancreatic collection measuring 9.1 cm x 3.8 cm

These laboratory findings were consistent with diabetic ketoacidosis (DKA). The patient was also severely hyponatremic (115 mmol/L), and the corrected sodium level was 122 mmol/L. He was then initiated on aggressive intravenous (IV) fluid resuscitation and insulin drip in the emergency room. He became more awake and alert with IV fluids, and upon further questioning, he complained of abdominal pain, nausea, and vomiting for the past five days. He could not provide any additional medical or family history except for a personal history of diabetes mellitus and stopping medications a year ago. A review of his chart revealed a prior admission two years ago, in June 2021, for hypertriglyceridemia-induced pancreatitis and DKA treated with insulin drip. Unfortunately, he left against medical advice at that time and was lost to follow-up. A computed tomography (CT) scan of the abdomen/pelvis was obtained, which revealed peripancreatic inflammation with associated necrosis and pseudocyst consistent with severe acute pancreatitis (Figure 1A). There was no evidence of cholelithiasis, choledocholithiasis, or biliary duct dilation. He denied any history of alcohol use. Other causes of acute pancreatitis were being evaluated. A lipid panel was obtained, and triglyceride levels came back significantly elevated at over 4,425 mg/dL (Table 3).

**Table 3:** Lipid panel on admission

Test	Result	Reference Range
Triglycerides	4425 mg/dL	39-160 mg/dL
Cholesterol	496 mg/dL	0-200 mg/dL
Low-density lipoprotein	100 mg/dL	<100 mg/dL
Very low-density lipoprotein	306 mg/dL	<30 mg/dL
High-density lipoprotein	11 mg/dL	35-55 mg/dL

The patient was admitted to the intensive care unit (ICU) for DKA and hypertriglyceridemia-induced acute pancreatitis; he was continued on IV fluids and insulin drip with close monitoring of electrolytes and triglyceride levels. General surgery was consulted to assist with a biopsy of the lesions for further evaluation of skin lesions, and the pathology report was consistent with eruptive xanthomas. A CT scan of the abdomen/pelvis was repeated to reassess the patient's pancreatitis with management thus far. It revealed peripancreatic fluid collection, which was stable compared to the initial CT scan (Figure 1B). The patient showed gradual clinical improvement, his DKA resolved, and his triglyceride level trended to 822 mg/dL in 48 hours. The CRP also trended down to 97 mg/L. He was taken off the insulin drip, started on subcutaneous insulin and fibrate therapy, and counseled on dietary modifications. The patient's hemoglobin A1c was significantly elevated at 12.5%, and he was also advised on medication compliance and tighter control of blood glucose levels. The patient's abdominal pain eventually improved; he tolerated the diet well and was discharged on insulin and fibrate therapy with recommendations to follow up with his primary care physician for close monitoring.

## Discussion

Acute pancreatitis occurs when an inciting event leads to inflammation of the pancreas. While the pathogenesis is not well understood and varies depending on etiology, early acute changes that result from the insult include activation of large amounts of trypsin that overwhelm the defense of autolysis, vascular injury/impairment, and the release of inflammatory mediators from granulocytes and macrophages [5]. Acute pancreatitis is one of the leading causes of hospital admissions from the emergency room, with acute pancreatitis accounting for 70% of pancreatitis cases (vs. chronic) in 2014 [1]. Among the many etiologies of acute pancreatitis, gallstones (35%-40%), alcohol use (30%), and hypertriglyceridemia (2%-4%) are the most common [2,4]. All patients diagnosed with acute pancreatitis secondary to hyperlipidemia had a mean triglyceride level of 4587 +/- 3616 mg/dL and presented with abdominal pain, nausea, and vomiting for hours to days. Their associated history was poorly controlled diabetes (72%), and they had a history of hypertriglyceridemia (77%) [4]. The risk of acute pancreatitis is correlated with serum triglyceride levels, and it was found that if triglyceride levels were over 1000 mg/dL and over 2000 mg/dL, there was approximately a 5% risk and 10%-20% risk of developing acute pancreatitis, respectively [3].

In our case, the patient's initial presentation was unique, which included generalized weakness and diffuse skin rash. His skin lesions, later found to be xanthomas, were an important diagnostic clue. Eruptive xanthomas are highly associated with serum triglyceride levels over 1500 mg/dL and hypertriglyceridemia-induced pancreatitis [6]. His other complaint of generalized weakness manifested as a result of DKA. Patients with DKA may present mentally obtunded, primarily when severe acidosis exists (a bicarbonate level of 4 mmol/L) [7]. Severe hyponatremia (115 mmol/L) is likely pseudohyponatremia associated with hyperglycemia and hypertriglyceridemia. Interestingly, his lipase levels are not significantly elevated, which is a rare event, and only very few cases are reported in the literature where patients had normal lipase levels in the setting of acute pancreatitis. Recent literature has reported a negative predictive value of serum lipase in diagnosing acute pancreatitis between 94% and 100% [8]. Amylase and lipase levels may remain normal if the destruction of acinar tissue during previous episodes precludes the release of sufficient amounts of enzymes, which might be the case in our patient [9].

Once IV fluids and insulin drip were initiated, the patient relayed his other concerns, including abdominal pain, nausea, and vomiting, in which prompt diagnosis of his condition was established. He was admitted to the ICU and started on an insulin drip and fibrate therapy. His triglyceride level decreased from 4425 mg/dL to 822 mg/dL in 48 hours. Ideally, for the management of hypertriglyceridemia-induced pancreatitis, the insulin drip should be continued until the triglyceride levels are below 500 [10]. Plasmapheresis should be considered in the presence of problematic features, including hypocalcemia, lactic acidosis, signs of worsening systemic inflammation or organ dysfunction, and/or multi-organ failure [11]. Additionally, the use of combined blood purification therapy, high-volume hemofiltration, and hemoperfusion can also be considered, depending on the severity of the acute pancreatitis [12].

To prevent recurrence, hypertriglyceridemia management is based on modifiable and/or unmodified factors. Modifiable aspects include diabetes and hypertension control and promoting an active lifestyle. Dietary modifications include reducing simple carbohydrates and saturated fats with an increased fish intake, which is high in omega-3 fatty acids [13]. Alcohol cessation is recommended as alcohol increases triglyceride levels with a risk of pancreatitis occurring [13]. These conditions involve discussing with the patient over time and building rapport with them for them to better themselves and change their behavior. In cases of unmodifiable hypertriglyceridemia or if the modifiable factors do not result in improvements, medications are used as well. Statins have a mild to moderate reduction of triglycerides, which can help reduce the risk of pancreatitis compared to the placebo [14]. When fibrates are used, fenofibrate is the better option compared to gemfibrozil as it has fewer drug-drug interactions, fewer side effects, and better patient compliance due to its once-daily dosing [14]. Our patient, once stable, was discharged on glycemic control with insulin in addition to fibrate therapy and advised strict follow-up with his primary care physician.

Another problem that is worth investigating further is the multiple episodes of hemolysis during his CMP collection. Blood samples can hemolyze either in vitro (associated with sample collection) or in vivo (premature death of red blood cells) [15]. His severe metabolic derangements (metabolic acidosis and hyperlipidemia) exacerbated this issue, warranting further investigations. Hemolysis is a rare complication of hyperlipidemia and likely occurs due to the destabilization of red cell membranes in the presence of lipemia [16]. The combination of unclear complaints, altered mental status, and issues during blood collection is a challenging clinical scenario that can delay diagnosis and treatment. Patients with a history of uncontrolled diabetes may benefit from prompt evaluation for emergent diabetic complications such as DKA and hyperosmolar hyperglycemic syndrome, especially if the presentation is not clinically obvious.

## Conclusions

Acute pancreatitis can result from many inciting events (e.g., gallstones, alcohol use, hypertriglyceridemia). Hypertriglyceridemia-induced pancreatitis commonly presents with abdominal pain, nausea, and vomiting with a history of uncontrolled diabetes mellitus and hypertriglyceridemia. Other emergent complications of diabetes mellitus (e.g., diabetic ketoacidosis or hyperosmolar hyperglycemic syndrome) may also further exacerbate the underlying condition. It is important to recognize dermatological conditions (e.g., eruptive xanthomas) associated with risk factors (e.g., hypertriglyceridemia) and disease processes, as it can lead to diagnosis and treatment, especially when history is limited. Proper follow-up with primary care physicians, adherence to medications, and healthy lifestyle changes can help prevent recurrent pancreatitis and reduce complications associated with pancreatitis.
